# Large-scale real-life implementation of technology-enabled care to maximize hospitals' medical surge preparedness during future infectious disease outbreaks and winter seasons: a viewpoint

**DOI:** 10.3389/fpubh.2023.1149247

**Published:** 2023-08-09

**Authors:** Talia Sener, Winne Haenen, Patrick Smits, Guy H. Hans

**Affiliations:** ^1^Faculty of Medicine and Health Sciences, University of Antwerp, Antwerp, Belgium; ^2^Federal Public Service for Health, Food Chain Safety and Environment, Brussels, Belgium; ^3^Cell Crisis Preparedness, Agentschap Zorg en Gezondheid, Brussels, Belgium; ^4^Chief Medical Officer, Antwerp University Hospital (UZA), Edegem, Belgium

**Keywords:** infectious disease, outbreak, hospital at home, surge capacity, digital health, remote care

## Abstract

Hospitals can be overburdened with large numbers of patients with severe infectious conditions during infectious disease outbreaks. Such outbreaks or epidemics put tremendous pressure on the admission capacity of care facilities in the concerned region, negatively affecting the elective program within these facilities. Such situations have been observed during the recent waves of the coronavirus disease pandemic. Owing to the imminent threat of a “tripledemic” by new variants of the coronavirus disease (such as the new Omicron XBB.1.16 strain), influenza, and respiratory syncytial virus during future winter seasons, healthcare agencies should take decisive steps to safeguard hospitals' surge capacity while continuing to provide optimal and safe care to a potentially large number of patients in their trusted home environment. Preparedness of health systems for infectious diseases will require dynamic interaction between a continuous assessment of region-wide available hospital capacity and programs for intensive home treatment of patients who can spread the disease. In this viewpoint, we describe an innovative, dynamic coupling system between hospital surge capacity and cascading activation of a nationwide system for remote patient monitoring. This approach was developed using the multi-criteria decision analysis methodology, considering previously published real-life experiences on remote patient monitoring.

## Introduction

An acute, highly infectious disease is caused by microorganisms including bacteria, viruses, parasites, or fungi. Depending on a patient's personal risk factors and immune system status, such acute infections can either have a relatively mild or a more severe course, resulting in health complications that can even cause death in worst-case scenarios. Patients generally remain at home during mild disease courses. However, if the infection is severe or when the clinical symptoms worsen over time, the patient will require hospitalization for follow-up and for more invasive management of symptoms or complications. During epidemics, such as the recent outbreak of severe acute respiratory syndrome coronavirus 2 (SARS-CoV-2), when many patients became infected and their number continued to rise over several weeks, the pressure on health systems, including hospitals, increased dramatically (1–3). This increased pressure was also transferred to the nursing and medical staff while they had already been chronically stretched to the limit in most countries because of a continuous scarcity of resources (4–6). Consequently, elective care had to be canceled as a countermeasure to preserve sufficient hospital capacity for newly presented patients with severe acute infectious conditions even when some hospitals tried to open additional acute beds (7–9). Further disruption seems inevitable in the future unless robust measures are urgently introduced (10). In a recent survey, 40% of hospital systems surveyed did not believe they would be able to return to historical procedural throughput levels even if demand increased to previous levels or higher (11).

However, as documented during the past 3 years, not all patients can be admitted to the hospital or receive specialized care during large-scale outbreaks owing to existing staff limitations (9, 12). The lack of staff within hospitals and other care facilities has been ongoing for a long time and cannot be solved overnight, if at all. We urgently need to implement innovative approaches in the near future to regulate the inflow of patients into our hospitals and safeguard sufficient hospital capacity for other acute and chronic pathologies, such as cancer treatment, surgery, care for older adults with frailty, and even complicated childbirth. All the above must be conducted while preserving safe and optimal care for patients who cannot be admitted to the hospital during outbreaks or epidemics. To safeguard hospital capacity for those in need, as many patients as possible should receive all the necessary diagnostic and therapeutic interventions within the outpatient setting to maintain the disease state under control, avoiding deterioration. It is also critical to consider that the actual available hospital capacity is lower than that on paper since many care institutions worldwide are forced to underutilize the available beds because of personnel scarcity. Rooms and “functional” beds should be available and staffed to be factored into the monitoring system.

The main problem we want to address in this viewpoint is to find a widely deployable solution to interconnect free hospital capacity with a larger-scale application of technology-enabled care during infectious outbreaks. We propose an innovative, dynamic coupling between hospital capacity and remote care programs to regulate the influx of patients within a large geographical area. We applied a decision analysis method incorporating multiple criteria to elaborate on this proposal. Based on the Belgian approach to tackling the coronavirus disease (COVID-19) crisis, the functioning of different government agencies overlooking the crisis, the number of available hospital beds during the different waves of COVID-19, and considering similar international projects, the authors explore the balance between the advantages and disadvantages of alternatives to preserving hospital capacity during future epidemics and supporting integrated care models in such challenging conditions. Digital health enabling integrated care can be defined as the use of digital health-related technologies to enable and support the functional activities and processes required to achieve the aims of such integrated models of care (13). This is often referred to as the Task-Technology Fit model, first described by Goodhue and Thompson (14). The proposed dynamic approach model was developed according to the described Task-Technology Fit model. During the creation of this dynamic model, we based ourselves on previous real-life experiences and applied all relevant information from these publications to our model. Relevant studies are listed below, although it is not our intention to provide a comprehensive review on the topics of technology-enabled care or the application of digital health in infectious diseases. By combining international experiences with our own experience during the COVID-19 crisis, we propose a new approach for future epidemics.

## Technology-enabled care as a new methodology for preserving open hospital capacity during outbreaks

A population-based introduction of technology-enabled care (TEC) facilities may provide a durable solution to manage the increased workload in hospitals during surges of infectious diseases. TEC is a collective term referring to the use of telehealth, telecare, telemedicine, telecoaching, and guided self-care for patients with both acute and long-term conditions (15). It should be convenient, accessible, and cost-effective (15–17). Furthermore, the process must be beneficial for all parties involved. Thus, TEC is increasingly considered a solution for the many challenges the health sector is currently facing. Digital health services foster innovations in home care, quality improvements in patients' lives, and provide much more efficient and effective solutions for healthcare providers (18). A recent review on patient experiences with TEC found that patients felt TEC had several advantages, such as offering greater empowerment, helping patients better understand their conditions, increasing awareness regarding symptomatology and treatment, being safer, and increasing self-efficiency (15). However, some patients felt frustrated and stigmatized when not able to use such digital solutions. The COVID-19 pandemic created a unique opportunity to explore TEC use in real-life clinical settings, and we should now further develop such services to be embedded into innovative generic pandemic preparedness programs.

The recent COVID-19 crisis also demonstrated that digital services could not only ameliorate physician workloads, but also offer patients a personalized health service (19–21). A cohort study in Galicia (Northwestern Spain) proved that proactive at-home telemonitoring of patients with COVID-19, even of those with a high risk for complications, was indeed associated with reduced pressure on hospital capacity and lower mortality rates (22). Another prospective observational study tracked high-risk patients with polymerase chain reaction confirmed COVID-19 (21); the results indicated that telemedicine with home telemonitoring was a clinically useful and secure method. Only 8% of these patients needed hospitalization, and no complications or deaths occurred at home. Another pilot study investigated the implementation of a home monitoring system for patients who were considered at significant risk for clinical deterioration (23). Supported self-monitoring at home proved to be safe and reassuring to patients. However, patients had to be protected against a false feeling of safety. It is, therefore, important that patients are prompted to continue measuring their vital signs repeatedly, also when feeling well overall, and that warning messages to patients contain the warning signs of asymptomatic hypoxia. A cohort study of 6,822 patients that retrospectively assessed patient responsiveness to a remote care program and clinical outcomes during the COVID-19 pandemic reported that individuals who engaged virtually were less likely to experience an emergency department visit, hospital admission, or admission to an intensive care unit (20). The Mid-Atlantic Academic Health System in the United States performed a retrospective cohort analysis of their automated text messaging service for monitoring COVID-19 at home (COVID Watch) (24). The program comprised twice-daily automated text message check-ins and an option to report worsening symptoms at any time. A total of 3,488 patients enrolled in the remote care program, and 4,377 in the usual care program. At 30 and 60 days, using COVID Watch led to fewer deaths per 1,000 patients, even after adjustments for differences in patients' clinical and demographic characteristics. One-third of the deaths in the usual care group occurred outside the hospital, and none occurred during inclusion in the remote care program. Patients in the remote care group had more telemedicine encounters, more emergency department (ED) visits, and presented to the ED sooner by a mean of 1.9 days. Another large-scale retrospective cohort study investigated the impact of remote monitoring on hospitalization outcomes in patients with COVID-19 (25). In total, 10,660 patients were eligible for inclusion, with a total of 5,364 patients who activated monitoring. After adjustments for demographics, comorbidities, and time, activation of remote care was associated with lower odds of hospitalization, a longer mean time between testing and hospitalization, a shorter length of stay, and less intensive care use.

During the winter of 2020–2021, remote home monitoring of people testing positive for COVID-19 using pulse oximetry was implemented across England (26). The main purpose of the COVID oximetry @home (CO@h) project was to identify falling blood oxygen saturation levels at an early stage, enable earlier hospital admission, reduce the need for intensive care, and improve survival. None of the results proved to be statistically significant, but findings indicated that for every 10% increase in coverage of the program, mortality was reduced by 2%, admissions increased by 3%, and lengths of stay increased by 1.8%. These findings may be somewhat disappointing at first glance, but several explanations are possible. The CO@h program was facing low rates of enrollment and incomplete data in many areas and was implemented in many ways across the country. This may have led to varying levels of impact on the outcome measures. During the COVID-19 crisis, several projects were developed to implement so-called “virtual wards,” providing ongoing treatment at home after hospitalization (27–30). No substantive conclusions can currently be reached regarding these virtual ward models because of the lack of standardized protocols, lack of uniform reporting, and missing data (30). Other programs focused on earlier discharge of patients with COVID-19 from the hospital by applying accelerated care pathways and post-discharge enrollment in remote patient monitoring (RPM) services (31, 32).

Several other approaches to applying TEC to maximize hospitals' medical surge preparedness were studied during the COVID-19 crisis, with some projects focusing on the application of virtual care in the outpatient care setting (33). The findings suggest that TEC use can decrease emergency room visits, safeguard essential healthcare resources, and positively affect the virus's spread. Other authors broadened the scope and investigated the eventual implementation of a nationwide emergency digital network for critical care (34). Such a network would be instrumented to support patients, increase healthcare capacity, and predict/prevent future infectious disease outbreaks. Jnr et al. presented practical approaches to ensure the quality of service for telemedicine consultations through the application of software-based networking (35). Such infrastructure should support healthcare professionals in overcoming issues arising from the congestion of conventional networks during outbreaks and disasters. In addition, proper national planning of digital health should be implemented to maximize its applicability (36).

Currently, the application of RPM programs for other infectious diseases than COVID-19 remains extremely limited. Concerning influenza, some earlier studies have focused on vaccination and providing rapid testing at home (37, 38). Previously, telehealth program use was proposed as a tool for early identification of pandemic influenza activity (39), but to our knowledge, no follow-up programs were developed for acute influenza-related infections or for the respiratory syncytial virus. A 2008 US retrospective study examined the impact of telehealth technology (mostly interactive video-conferencing) in providing timely, efficient, and prudent care for patients in rural areas on three commonly occurring infectious diseases: community-acquired bacterial pneumonia, febrile neutropenia, and skin-wound infections (40). Patients treated via telehealth had fewer days on antibiotics and fewer days of hospitalization than patients treated via in-person intervention (standard of care). Survival rates did not differ significantly between the groups. Furthermore, telemedicine has been investigated in disease surveillance in rural areas, where delays in disease recognition and intervention can lead to the uncontrolled spread of infectious diseases, or to the more rapid prediction of failure of installed therapy in such areas (41–43). Finally, blockchain-enabled digital health passports have been proposed for use in situations where contact tracing of infectious patients can be crucial (44).

## Real-life implementation of a remote care program: a Belgian experience

As observed during the COVID-19 pandemic, hospital conglomerates developed and managed most TEC-based programs. In Belgium, the TeleCovid program was developed by our team within the Antwerp University Hospital (https://www.telecovid.be). Between January 2021 and September 2022, a total of 435 patients with COVID-19 were included in this program (45), with 2.07% of patients requiring hospitalization. One patient died during hospitalization while no deaths occurred at home. After September 2022, patients with multiple infectious diseases such as monkeypox, influenza, and COVID-19 were included in the program (*n* = 89; until March 30, 2023). To enable a roll-out of this RPM program at a national level, we implemented a 3-pillar approach, consisting of a logistical back office (“dispatching”), a medical team taking care of the continuous monitoring of medical conditions (“monitor team”), and an IT helpdesk to support patients in accessing the digital platform and coupling the telemetric devices. All three teams had to be able to handle large numbers of patients on a 7/7 basis and display the flexibility to tackle all types of unexpected challenges. Dispatching ensured that patients could be quickly included in the TeleCovid RPM. Patients could be registered initially by primary care physicians, emergency physicians, or other medical specialists with whom the patients have an established care relationship. To facilitate a smooth registration process, a dedicated telephone number had been installed (operated all week), as well as a dedicated secure form on the hospital's website. In 89% of the cases, the TeleCovid care pathway was activated within 6 hours after initial registration. After activation, patients were asked to provide their informed consent regarding participation in the program. Next, a risk stratification questionnaire was activated for the patients to assess their personal risk profile upon entering the remote care program. The mean duration of participation within the virtual care pathway was 11.6 days. Follow up consisted of diaries investigating the presence and intensity of a large variety of symptoms, validated questionnaires (focused on quality of life and functionality), and measurement of vital signs using medical-grade telemetric devices. All devices were Bluetooth-operated and exclusively linked to the patient's mobile phone (by encoding a unique box code in the device), so automated data transmission was secured at the highest level. The dedicated secured platform and app (UZA@home^®^) also incorporated a two-way communication tool to make low-threshold communication possible between patients and the remote care team. Secured transmission of clinical pictures was possible, so the evolution of rash or COVID-19 toes could be closely monitored by the remote care team. Video consultations could be easily scheduled from within the platform, allowing up to three caregivers to interact with the patient simultaneously. Furthermore, the dispatching team had the important task of coordinating the needed care and gathering all necessary healthcare providers (HCP) and supporting facilities around the patient, always in close cooperation with primary care facilities. For instance, dispatching often kept contact with pharmacists to provide the proper medication to eligible patients. If necessary, they also linked patients to social workers to provide support to them and their family caregivers. In contrast, the medical monitor team focused on monitoring the clinical condition, safeguarding a smooth flow of medical information between all concerned HCP, and initiating all necessary therapeutic interventions within the setting of a home environment (e.g., medication, oxygen therapy, blood sampling, wound care, physiotherapy). In case of deterioration in the clinical condition, short communication lines were established with primary care physicians to notify them. The final decision for eventual hospitalization, in the TeleCovid project, was determined by the family physician, who was most familiar with the history and co-morbidities of the patients and had the best knowledge concerning the possible care provided by the social environment. Furthermore, the medical monitoring team was closely connected with emergency facilities, so urgent pick-up and transportation could be provided upon request. In that situation, the monitoring team would identify the nearest suitable hospital, provide the briefing of the receiving hospital teams, and take care of the immediate transferal of patients to the appropriate wards or monitoring units, so that no additional time would be lost on the emergency wards. In its operationality, the TeleCovid program showed significant similarities with the “Home Test to Treat” program of the US National Institute of Health (NIH).

Owing to the increasing availability of specific antiviral treatment options, accessibility to home administration of such drugs became essential since the beginning of 2022 and will undoubtedly become even more critical during future outbreaks. Antiviral drugs should be quickly provided to high-risk patients to minimize the severity of the infection and ameliorate the clinical condition. Fast-tracked electronic drug prescription was included in our TeleCovid protocol. A mobile service by home nurses was used to deliver the drugs at home since many patients were unable to go to the pharmacy. Telemetric devices were delivered during the same visit. Nurses seized this opportunity to assess the patient's clinical condition and ensure the proper initiation of virtual measurements of vital signs. However, home-based antiviral therapies can be challenging when applying traditional care models; therefore, RPM can provide a significant advantage in comparison to the current standard of care. High-risk patients often have various underlying comorbidities for which they receive maintenance therapy, which can interact with antiviral drugs. Drug-drug interactions can decrease or increase blood serum levels, potentially leading to toxic syndromes. Therefore, it is vital for the medical monitor team to check for drug-drug interactions before initiating such therapies and take pragmatic measures such as adjusting doses or pausing comedication if necessary (46). Due to the aforementioned medical challenges, primary care providers are often reluctant to initiate such therapies.

The experiences from TeleCovid, and those from other international programs, indicated that remote care teams can take the lead in such circumstances through the integrated application of a full set of clinical and non-clinical monitoring services, such as telemetric devices with automatic transmission of readings to a hub, alarm activations, application of software algorithms for processing gathered information, back-up by specialists, and low-threshold bidirectional digital communication with patients. In addition, the remote care team also needs to closely monitor the eventual appearance and severity of drug-related side effects when anti-viral therapies are initiated at home, along with the objective clinical signs associated with the infectious disease. The uniqueness of the TeleCovid program lies in the fact that its mode of action is not limited to merely the functionality of a clinical command center—monitoring patients remotely and responding to alarms that are detected—but that it also provides a personalized and comprehensive approach to all (para)medical problems patients are faced with during the follow-up period.

## Implementing generic remote care programs during outbreaks: requirements and proposed modalities

Large-scale application of RPM could prove essential for safeguarding the integrity of first and second lines of healthcare in such high-demand circumstances. Despite evidence of its clinical benefits (47), the widespread implementation of remote symptom monitoring remains limited. Core components of remote monitoring programs should include electronic delivery of diaries and surveys with actionable symptoms, patient education, system monitoring compliance and symptom severity in real-time, and capacity to generate alerts and identify personnel responsible for follow up, along with management of alerts and symptoms (48). Questionnaires related to patient symptoms and their severity should be monitored daily. The use of diaries makes it possible to quickly map additional information on newly emerging symptoms outside the questionnaire's scope and monitor them in detail. Telemetric medical-grade devices, such as blood pressure monitors, digital thermometers, and peripheral oxygen saturation meters, can be quickly delivered to patient home addresses and be used for intensive objective surveillance of patients' symptomatology over longer periods. Moreover, easy-to-use digital communication should be established between the monitoring team and patients (or relatives). In this way, a secure environment can be created in which healthcare professionals will monitor the patient intensively, and the home stay of the patient will be guaranteed in safe conditions. However, it is currently not feasible to include all patients with infectious diseases within a population in such remote monitoring programs. Therefore, difficult choices must be made to identify patients to closely monitor at home during their acute infectious disease state. Three parameters are of paramount importance in making these decisions: patient risk profile, their tech savviness, and simultaneous hospital occupancy.

As hospital capacity becomes compromised during infectious outbreaks, healthcare professionals must become more restrictive regarding the influx of additional patients with infectious diseases. In such circumstances, the health authorities should maximize the availability and capacity of remote care programs. When hospital occupancy decreases again, more patients can be readmitted to the hospitals, and home-based monitoring can be restrained. Such flexible inclusion criteria should be based on the risk profiles of individual patients to avoid discrimination based on social or economic factors.

Depending on the pathogen causing an increase in acute infections, high-risk patient populations can be identified based on international guidelines for recent infectious pathologies (see Table 1). Identification of high-risk populations should be closely monitored and periodically adapted by an independent medical council. Patients with higher disease-related risk factors are at a far greater risk of hospitalization owing to their deteriorating medical condition. Patients in a high(er)-risk group or with existing comorbidities often have difficulty clearing their body from the pathogen. In the past diverse risk factors have been identified with a negative impact on the disease course of infectious diseases in selected patient populations. Pre-existing physical conditions such as chronic kidney disease, cardiovascular disorders, diabetes, history of transplantation, and immunosuppressant disorders (49–54). Mental conditions can also negatively influence the disease course in infectious patients (55, 56). But also lifestyle variables can negatively impact the outcome of infectious diseases (57, 58).

**Table 1:** Patient-related risk factors that could cause a patient to become seriously ill from an acute infectious disease, like SARS-CoV-2, Influenza, Respiratory syncytial virus (RSV), and Monkeypox (MPX).

**Risk factors for hospitalization**	**SARS-CoV-2**	**Influenza**	**RSV**	**MPX**
	Immunocompromised patients			
	Older frail adults (> 80 years)	Older adults (>65 years) Pregnant and postpartum (up to 2 weeks after partus)	Infants, especially premature infants, or babies < 6 months Older adults (> 65 years)	Children (mainly < 8 years) Pregnant and breastfeeding women Individuals with one or more complications caused by the infection
	Cardiovascular diseases Chronic lung diseases Neurological and cognitive disorders Hematological disorders Endocrine disorders: diabetes Chronic kidney disease Obesity		Adults with chronic (congestive) cardiovascular diseases or chronic (obstructive) pulmonary diseases	
	Hematological cancers Solid cancer	Liver diseases Metabolic disorders		

## Interaction between free hospital capacity and remote care: a proposed dynamic coupling model

The rollout of such large-scale RPM should be triggered by the available hospital capacity within the affected region. Hospitals' medical surge preparedness or surge capacity plays a significant role in reducing mortalities in case of disasters and emergencies (59–61). Several models were developed including transportation of patients from one facility to another hospital or even relocating surgical services to satellite hospitals to increase theater and critical care capacity (2, 60, 62, 63). As in several other countries, a hospital contingency plan has been implemented in Belgium since the start of the COVID-19 pandemic (64–66). The Belgian hospital contingency plan was proposed by the Hospital Transport & Surge Capacity Committee. This advisory body recommends taking adequate control measures for hospitals and patient transport capacity. The plan consists of different phases, with a continuous and dynamic evaluation of hospital occupancy, and it sets the percentage of beds each hospital needs to keep available in case of a new surge of infectious disease. In Phase 0, 2.5% of the acute beds and 15% of the intensive care unit (ICU) capacity must be made available for a new surge. In Phases 1A and 1 B, 5 and 7.5% of the acute beds, respectively, must be exempted (and 25–33% of the ICU capacity). In Phases 1C and 1D, 10 and 12.5% of the acute beds and 50–60% of the ICU capacity must be kept available for patients with infectious diseases, respectively. The current hospital surge capacity plan tries to avoid opening additional hospitals or ICU beds since past experiences have shown that the opening of additional hospitalization capacity was linked to an increase in mortality (67, 68).

**Figure 1:**
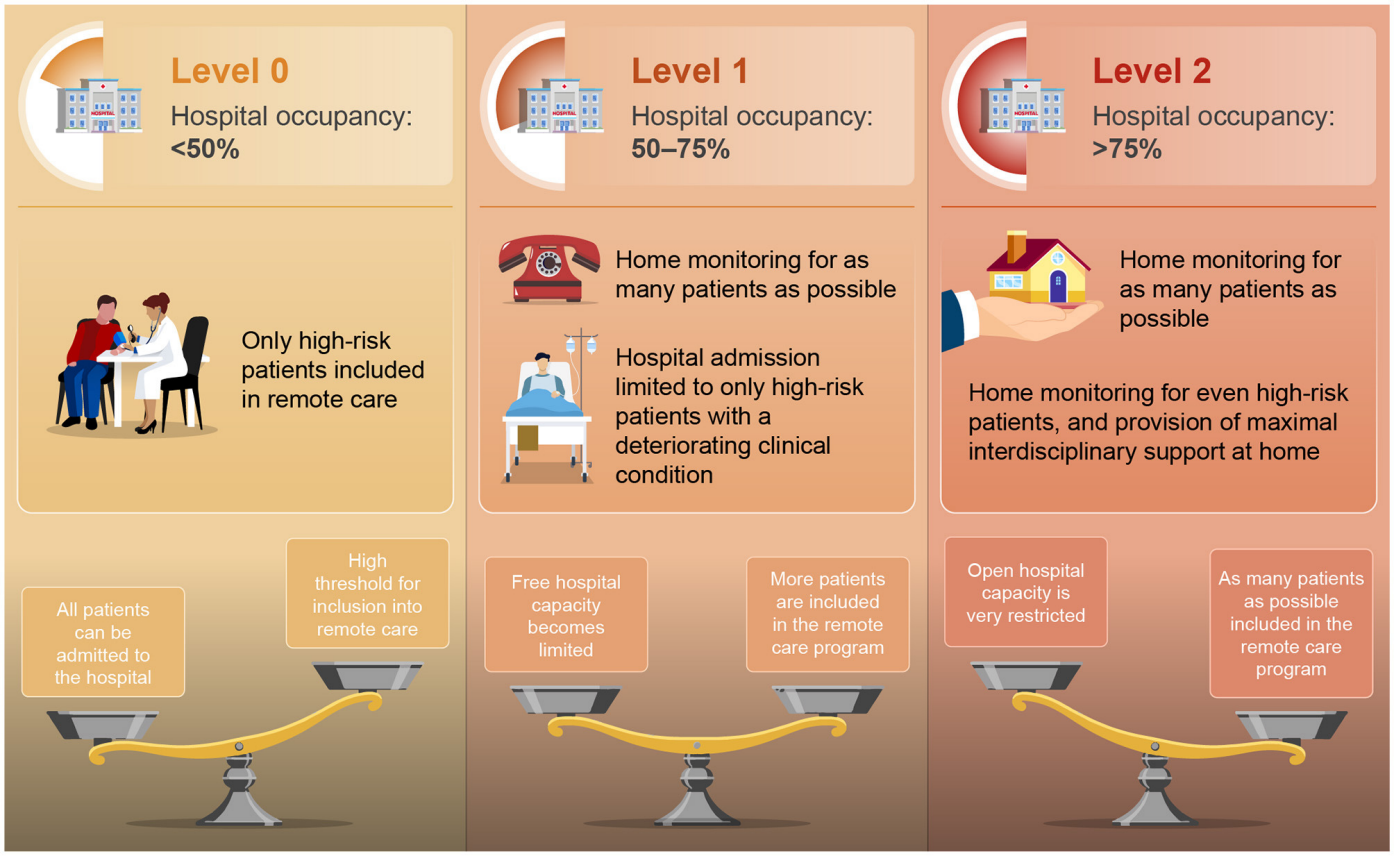
Proposed dynamic link between hospital surge capacity and nation-wide application of technology-enabled care. Restricted use of remote care to high-risk patients in level 0, to a much broader inclusion of patients in remote care program in level 2 (very limited availability of free hospital beds).

The current plans, therefore, focus on an optimized use of available capacity. However, activating such surge capacity will inevitably result in fewer beds available for all other non-infectious pathologies, negatively impacting the entire spectrum of planned care within a geographical region. Based on past experiences, the authors developed a new paradigm was developed using a multiple criteria decision analysis (MCDA) (69, 70). At first, the objective was defined. As the number of patients with infectious diseases increases in hospitals, it becomes crucial to limit additional inflow as much as possible to avoid complete disruption of the healthcare system within that region. Only high-risk patients with severe infectious disease should be hospitalized. Treating as many patients as possible in their home environments is vital in such circumstances. Next, appropriate scenarios for optimizing organizational impact were explored. Elements such as hospital occupancy, the number of free beds, the reproduction number of the infectious pathogen(s), and the percentage of HCP on sick leave were hereby considered. At last, the final model and linked outcome measures were identified. Healthcare authorities should partner with hospital administrations to implement a dynamic system that models both caseload and hospital capacity requirements in real-time during infectious outbreaks or winter seasons with circulating viruses. If surge capacity is activated, it would automatically result in an adjustment of the inclusion criteria for remote home-based care programs. It should be noted that hospital occupancy of 85% is generally considered the limit at which hospitals can work safely and effectively (71). The proposed link between Hospital Surge Capacity staging and home monitoring application is described below and is shown graphically in Figure 1.

■ Level 0: Hospital occupancy < 50%.

° Hospital beds are widely available.

■ Threshold for monitoring patients with acute infectious disease at home is set as high as possible.

• Only high-risk patients are included in remote care (multiple risk factors present).

■ All patients can be admitted to the hospital when they fall too ill.

■ Levels 1A and 1B: Hospital occupancy >50%, but < 75%.

° Free hospital capacity becomes less available.

■ Threshold for home monitoring is lowered to patients with a more moderate risk profile.
■ Initiate home-monitoring in as many patients as possible, and limit hospital admission to only high-risk patients with a deteriorating clinical condition.

■ Levels 2A and 2B: Hospital occupancy >75%.

° Open hospital capacity is very restricted, with only 10% left until full capacity.

■ Threshold for inclusion into the home-care program should be as low as possible, including patients with a low-risk profile.
■ Monitor as many patients as possible at home, including high-risk patients, and provide maximal interdisciplinary support in their home environment.

## Discussion: challenges and limitations of the proposed methodology

The proposed approach provides in our opinion some unique advantages. The approach differs significantly from previously published digital-care programs in several aspects of elaboration and implementation. First, the proposed model is flexible in its practical implementation, ranging from limiting its activation to very selected patient populations to extending the influx of remote care to large-scale populations. Second, its deployment is determined on a national level so we can generate a substantial impact on free hospital capacity. Third, the proposed approach is generic and not limited to a single disease entity. This genericity allows application in various clinical circumstances and outbreaks. Fourth, the proposed use of TEC strongly aligns with the activities of first-line care providers and hospitals to add value and quality to the care provided. This is in accordance with the Task-Technology Fit model as described by Goodhue and Thompson (14). Finally, the proposed notion of the value of our methodology extends beyond individuals and processes but supports normative and functional changes at the individual, organizational, and societal levels. Thus, the proposed approach can be a crucial factor in the ongoing transformation of global health systems. The methodology is therefore disruptive in its approach, both by the described national extent as well as by the conception of the RPM program which is highly personalized and interactive. Future implementation studies will need to identify its value and practicality in diverse clinical circumstances.

Indeed, many drawbacks and challenges remain. The adoption of digital health tools should be closely monitored to ensure that the program we design today leads to greater integration tomorrow. The proper development and large-scale implementation of risk-stratification tools should be further investigated. One of the disadvantages is that TEC implementation can exacerbate health inequity at the individual level due to unequal digital literacy. Health equity should be conserved by making interfaces and workflows as simple as possible and supporting new users and communities to adapt to the new tools and become engaged (72). In addition, programs should incorporate flexible and multi-channel human-to-human communication pathways for handling more complex interactions or when patients are less confident in using high-tech devices. This will also offer a personal and empathic touch to the follow-ups that patients often crave. Values of equity, person-centeredness, and a comprehensive team-based approach should become the key drivers of integrated models of digital care while prioritizing patient experience and social determinants of health (73). Patients' experiences relate not only to the practical (technical) elements of the provided solution but also to how this impacts their everyday life (15). Patient participation in the development and planned use of such solutions is strongly advised. In addition, health agencies should require RPM uniformity to avoid having a large diversity of programs applying slightly different methods. Such uniformity will also positively impact both the quality and the security of the systems applied. The current lack of agreed standards for data protection, privacy, and security of both data and devices should be tackled to unlock the full potential of TEC. Stakeholders should build trust by developing strong privacy and security arrangements, adopting key principles of data minimization, data protection by design and default, and implementing data encryption and authentication mechanisms. Therefore, it is also crucial that data from such platforms are shared with electronic health records. In addition, the quality of the patient-generated health data (PGHD) must be secured. PGHD are collected continuously under the patient's responsibility in rapidly changing circumstances during the patient's daily life. This poses risks to the quality of PGHD and, in turn, reduces their trustworthiness and fitness for use in clinical practice. Recently a guideline was developed describing a systemic approach to data quality management of PGHD so that these data can be reliably used in clinical care (74). In the future, special attention should be given to the cost-effectiveness of such models and their sustainability. Economic analysis is currently not reported for most models and does not go beyond the simple reporting of resources used and the amount spent per patient monitored (30). A recent publication stated that RPM could further optimize hybrid in-home and remote nurse or physician evaluations, reducing costs by up to an estimated 3.5% overall (75).

The large-scale focus of the proposed methodology is quite different from previous experiences, so the expectations are set very high. Infectious disease states and free hospital capacity need to be monitored continuously through surveillance programs, and rapid action should be undertaken when significant changes in hospital phases are detected. RPM program should be on stand-by continuously, ready to be scaled up when necessary. In addition, the content and organization of hospital-level care at home should be adjusted quickly based on the characteristics of the prevalent infectious pathology. Methods of rapid identification of high-risk patients need to be available at the population level since they are essential for the roll-out of the different phases of the proposed methodology. All of this means that the implementation of RPM for infectious diseases will move from a project-based approach to a full-scale implementation in daily clinical practice. A close cooperation between national and regional health authorities, hospital systems, and first-line healthcare delivery systems is therefore mandatory.

Finally, technology is great but only when it works well. It is critical that the technologies involved work accurately, without glitches, and ultimately improve the quality of patient care provided.

## Conclusion

In this viewpoint, we propose an integrated approach where the application of technology-enabled care coupled with continuous monitoring of available hospital capacity in a region should support the application of a more integrated model of care as well as preserving capacity during future outbreaks. Theoretically, a digital monitoring program could include any patient infected by an infectious pathogen. However, from a broader public health perspective, it is crucial to include patients who are at an increased risk of a potentially disastrous course of their infectious disease if they are unable to be hospitalized. By establishing evidence-based risk factors for each endemic pathogen, healthcare providers can quickly identify patients at risk of future deterioration in their clinical condition and prioritize their inclusion in remote care programs, considering the real-time hospital capacity at that specific moment. With the increasing availability of antiviral and antibacterial treatment options that can be administered at home, the application of home-based pharmaceutical interventions will become vital in the development of future remote care programs.

Implementing such remote care programs in a broad geographical setting can help regulate hospital inflow during times of capacity scarcity while providing safe follow up and maximized treatment in a trusted home environment. On the other hand, the availability of such programs can reduce the length of stay, freeing up additional hospital capacity.

## Author contributions

TS gathered all necessary data and wrote a first draft of the paper. GH supervised and assisted in the writing of the different draft versions as well as the accuracy of the references. WH and PS contributed equally to the paper's content and revised the article's draft versions. WH conceptualized the proposed hospital surge capacity staging. PS further conceptualized the package of tasks to be performed within the remote care plan. All authors contributed to the article and approved the submitted version.
